# Application of Three-Dimensional Arterial Spin Labeling Technique in the Assessment of Cerebral Blood Perfusion in Patients with Middle Cerebral Artery Occlusion: Analysis of Clinical Implications and Prognostic Factors

**DOI:** 10.1155/2022/6990590

**Published:** 2022-08-10

**Authors:** Jianguo Zhou, Dayong Fu, Yun Meng, Mingcong Lu, Fangyun Hu, Hongke Cheng

**Affiliations:** ^1^Department of Radiology, Lianyungang Hospital of Traditional Chinese Medicine, Lianyungang, 222004 Jiangsu, China; ^2^Department of Radiology The Fourth People's Hospital of Lianyungang, Affiliated Hospital of Nanjing Medical University Kangda College, Lianyungang, 222000 Jiangsu, China

## Abstract

**Objective:**

To explore the value of three-dimensional- (3D-) arterial spin labeling (ASL) technique in evaluating cerebral perfusion in patients with unilateral middle cerebral artery occlusion (MCAO) and to observe the influencing factors of poor prognosis via long-term follow-up of patients who survived the disease.

**Methods:**

The clinical data of 60 patients with unilateral middle cerebral artery (MCA) M1 segment occlusion diagnosed by magnetic resonance angiography (MRA) from January 2018 to January 2022 were retrospectively analyzed. All patients were examined by routine MRI, MRA, and 3D-ASL, in which two postlabeling delays (PLDs; 1525 ms and 2525 ms) were used in 3D-ASL. Cerebral blood flow (CBF) in the regions of interest (ROIs) of MCA on the affected side and the mirror side was measured. The clinical data and laboratory indexes of patients were collected and evaluated by clinical scales. With the modified Rankin Score (mRS) as the outcome indicator, patients were assigned to either the poor or the good prognosis group to analyze the factors influencing patient prognosis via univariate and multivariate analyses.

**Results:**

Among unilateral MCAO patients, there was a significant difference in the CBF of the affected side between the PLD 1525 ms and 2525 ms groups (*P* < 0.05), but there was no significant difference in the CBF of the mirror side (*P* > 0.05). Compared with the mirror side, 43 cases (71.7%) of the affected CBF presented with hypoperfusion, 9 cases (15.0%) with normal perfusion, and 8 cases (13.3%) with hyperperfusion. Age, NIHSS score, collateral circulation, and homocysteine (Hcy) were identified by multivariate Logistic regression analysis as independent risk factors for adverse outcomes.

**Conclusion:**

MCAO can lead to cerebral blood perfusion decline, and 3D-ASL technique can evaluate the post-MCAO cerebral blood perfusion level. Old age, high NIHSS scores, poor collateral circulation, and high Hcy levels are associated with poor clinical outcomes.

## 1. Introduction

Stroke is the prime reason for death and disability worldwide, which can be broadly divided into ischemic stroke and hemorrhagic stroke [1, 2], the latter of which includes cerebral hemorrhage and subarachnoid hemorrhage, while the former is defined as an infarction of the brain, spinal cord, or retina [3]. Stroke, which is primarily attributed to atherosclerosis-induced arterial stenosis or occlusion, ranks second among all causes of disease deaths in the world with a prevalence on the rise year by year [4]. In China, stroke has become the third leading cause of death. Asians, Africans, and Spaniards have a higher prevalence of intracranial arterial stenosis than Caucasians, a study found by [5]. In Asian populations, stenosis or occlusion of intracranial arteries, especially the middle cerebral artery (MCA), is the main problem [6]. Impaired cerebral perfusion (CP) is one of the major pathological bases of ischemic cerebrovascular disease and is closely related to the clinical manifestations and severity of the disease [7].

Occlusion of the main MCA can lead to large-scale infarction in its blood supply area, namely, malignant MCA infarction, which can lead to poor prognosis and high mortality. The clinical presentations are contralateral hemiplegia, hemiparesthesia, homonymic hemianopia, and global aphasia, accompanied with or without severe disturbance of consciousness and spasms, and cerebral hernia due to brain edema and intracranial hypertension [8, 9]. CP is not only affected by the degree of vascular stenosis but also related to collateral circulation (CC) [10]. When ischemic events occur due to blood supply insufficiency caused by hypoperfusion and other inducements, effective CC opening can rapidly improve cerebral blood flow (CBF) perfusion in the ischemic area, thereby protecting ischemic brain tissue. Therefore, the accurate evaluation of postocclusion CP level is particularly important.

Arterial spin labeling (ASL) perfusion imaging technology [11] is a new magnetic resonance imaging (MRI) method for evaluating CBF perfusion in recent years. It adopts fast spin echo technology and uses water molecules in arterial blood in vivo as endogenous magnetic tracers for volume imaging, which can reflect microvascular perfusion level with high image signal-to-noise ratio (SNR) and small artifacts. At the same time, it is simple to operate and noninvasive without the need for exogenous contrast agent, allowing it to quickly and accurately identify the abnormal perfusion area and quantitatively analyze the regional CBF [12, 13]. ASL does not involve radiation or contrast agents, allows easy registration with structural MRI, and provides absolute CBF that has been shown to be reproducible across time scales from minutes, hours, to days [14, 15]. Bulder et al. [16] applied ASL technique to studying CP in young stroke patients and found that more than half of them had insufficient perfusion in the blood supply area of MCA and hypoperfusion in the symptomatic cerebral hemisphere. However, there is currently relatively little research on the application of it in patients with subtotal cerebral vascular occlusion. A recent trend in ASL development is the use of fast three-dimensional (3D) sequences, usually combined with background suppression, to improve the signal-to-noise ratio and reliability of ASL perfusion measurements [17, 18]. Accordingly, in this study, 3D-ASL technique was used to investigate the alterations of CBF in the cerebrovascular responsibility area of patients with unilateral MCA total occlusion, and long-term follow-up was conducted to observe and analyze the influencing factors leading to poor prognosis, so as to objectively evaluate the CBF perfusion level and blood flow reserve of patients with unilateral MCA total occlusion and provide theoretical basis for guiding clinical treatment and improving patients' prognosis and life quality. The research results are reported as follows.

## 2. Data and Methods

### 2.1. Research Participants

The present study retrospectively analyzed all clinical data of 60 patients (male-to-female ratio: 34 : 26, age: 55.60 ± 6.93) with unilateral MCA M1 segment occlusion diagnosed by magnetic resonance angiography (MRA) from January 2018 to January 2022. Inclusion criteria are as follows: (1) initial presence of transient ischemic attack or acute or chronic cerebrovascular disease that was diagnosed by imaging examination as unilateral MCA M1 occlusion [19], (2) complete cranial imaging and clinical pathological data, (3) none clinical therapeutic intervention before examination, and (4) complete follow-up data. Exclusion criteria are as follows: (1) patients with bilateral MCA stenosis; (2) brain tumor, inflammation, vascular malformation, cerebral trauma, or cerebral hemorrhage; (3) imaging examination showed signs of cerebral hemorrhage; (4) carotid stenosis or occlusion; and (5) incomplete clinical data. All procedures of this study were certified by the Ethics Committee of the hospital to meet the ethical requirements.

### 2.2. Data Collection and Inspection Methods

#### 2.2.1. General Data

Patients' general clinical data, including name, sex, age, past history, personal history, family history, neurological function score evaluated by the National Institute of Health Stroke Scale (NIHSS) [20], and clinical biochemical indicators, were collected. Primary outcome: the modified Rankin Score (mRS) [21], which was evaluated in January 2022 during the follow-up, was used as the outcome indicator. Taking mRS as the evaluation index, patients were assigned to either the poor (mRS > 2) or the good (mRS ≤ 2) prognosis group. The NIHSS scale mainly includes the level of consciousness, consciousness instruction, gaze, facial paralysis, lower limb movement, sensation, dysarticulation, and other related aspects, with a total score of 0-45. The higher the score, the more serious the neurological impairment.

#### 2.2.2. Inspection Methods

GE Discovery 750 3.0T superconducting MR scanner and 32-channel phased array surface coil were used for inspection. The patient was placed in the supine position, and a whole-brain MR scan was performed with the scanning baseline parallel to the anterior-posterior commissural line of the corpus callosum. Scanning sequences included routine sequences (sagittal and axial T1WI, axial T2WI, and axial T2 FLAIR), and three-dimensional time-of-flight MRA was performed. 3D-ASL imaging was performed using pulsed arterial spin labeling sequences, with the anterior-posterior commissural line as the scanning baseline and the scanning parameters set as follows: TR 4632 ms (postlabeling delay (PLD) = 1525 ms), TR 5327 ms (PLD = 2525 ms), TE: 10.5 ms, layer thickness: 4 mm, layer spacing: 0 mm, FOV: 24 × 24, and excitation times: 3. The PLDs used in this study were 1525 ms and 2525 ms. The scanning range was consistent with DWI structural image, covering from the vertex to the skull base. Then, circles of the same size were placed in the regions of interest (ROIs) in a mirror symmetric way, and the CBF value of the corresponding region was measured. The scanning time was 4 minutes 45 seconds and 5 minutes 09 seconds, respectively. After scanning, the obtained original images were transmitted to ADW4.7 workstation, and the whole brain perfusion CBF map (PLD = 1525 ms, 2525 ms) was automatically generated by the ReadyView software (FuncTool; GE Medical Systems). The ROIs were then manually delineated at the maximum level of the abnormal perfusion area by two associate chief neurological physicians using a double-blind method, and the mirror area was delineated to measure its CBF value by the mirror technique. The relative cerebral blood flow (rCBF; affected side/mirror side) and the ratio of the CBF of the ipsilateral to the mirror side were calculated, with a ratio of <0.9 being hypoperfusion (<0.5 being severe ischemia), 0.9-1.1 being isovolumic perfusion, and >1.1 being hyperperfusion.

#### 2.2.3. Cerebral CC Score

Two associate chief neurophysicians assessed the degree of CC opening according to the modified American Society of Interventional and Therapeutic Neuroradiology/Society of Interventional Radiology (ASITN/SIR) CC grading system [22], with the specific grading as follows: grade 0, in any period, there is no or only a very small amount of leptomeningeal collaterals (LMCs) (less than 50% blood flow compared to the normal side) in the ischemic area; grade 1, partial CC (50%-100% CBF compared to the normal side) is not seen in the ischemic area until the late-venous phase; grade 2, partial CC (50%-100% CBF compared to the normal side) in the ischemic area can be seen before the venous phase; grade 3, complete CC in ischemic area can be seen in the late-venous phase (100% CBF compared to the normal side, whether or not partial collateral vessels are found before the venous phase); grade 4, complete CC (100% CBF compared to the normal side) can be seen prior to the venous phase. Grades 0-2 were considered as poor CC, and grades 3-4 were considered as good CC.

### 2.3. Statistical Processing

All data were statistically analyzed by the SPSS 22.0 software (IBM Corporation, Armonk, NY). Normally distributed continuous variables were described by the mean ± standard deviation, while nonnormally distributed ones were denoted by the median (lower quartile and upper quartile). The categorical data were presented as *n*(%), and chi-square test was used. In the univariate analysis, the continuous variables conforming to normal distribution and skew distribution were compared by independent sample *t*-test and Mann–Whitney *U* test, respectively. Using mRS as the outcome indicator, cases were assigned to two groups to analyze factors influencing long-term prognosis by multivariate Logistic regression. *P* < 0.05 was the significance threshold.

## 3. Results

### 3.1. CBF of the Affected Side and Mirror Side in Patients with Unilateral MCAO

**Figure 1:**
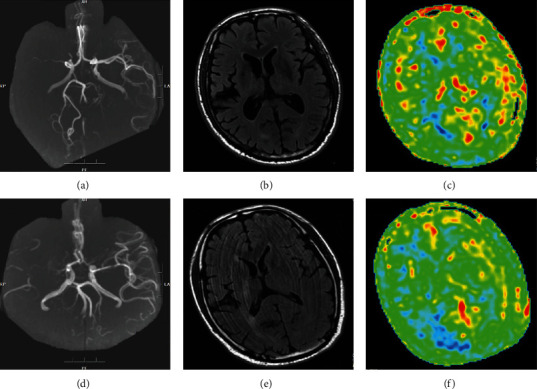
Regions of interest in the blood supply region of two patients' brains. (a) MRA indicated occlusion of M1 segment of the right MCA; (b) DWI showed negative; (c) ASL suggested that the CC was well established (PLD 2525 ms); (d) MRA showed that the M1 segment of the right MCA was occluded; (e) DWI showed negative; (f) ASL indicated insufficient collateral circulation (PLD 2525 ms).

In all patients with unilateral MCAO, there was no significant difference in the CBF value of the brain tissue in the blood supply area of the mirror side between the 1525 ms group and the 2525 ms group (*P* > 0.05), but there was significant difference in the CBF value of the affected side between the two groups (*P* < 0.05). CBF values of the brain tissue of the mirror side and the affected side were significantly different (*P* < 0.05, Table 1). The imaging data of two typical cases are shown in Figure 1.

### 3.2. Perfusion Status of the Affected and Mirror Side of Patients with Unilateral MCAO

Compared with the mirror side, the rCBF of the affected side showed hypoperfusion (the CBF of the affected side was significantly lower than that of the mirror side) in 43 cases and normal perfusion in 9 cases, with significant difference in the CBF between PLD 1525 ms and 2525 ms groups (*P* < 0.05). Hyperperfusion of rCBF was observed in 8 cases, but there was no significant difference in CBF of hyperperfusion between PLD 1525 ms and 2525 ms groups (*P* > 0.05, Table 2).

### 3.3. Baseline Data of Patients in Different Functional Prognosis Groups

The poor prognosis group had obviously higher age, NIHSS scores, and homocysteine (Hcy) levels while lower triglyceride levels than the good prognosis group, with statistical significance (*P* < 0.05). Besides, a significant difference was determined in the proportion of CC between groups (*P* < 0.05, Table 3).

### 3.4. Multivariate Logistic Regression Analysis

The variables with significant differences in univariate analysis in 2.3 were taken into multivariate Logistic regression analysis. Through multivariate Logistic regression analysis, it was found that age, NIHSS score, CC, and Hcy were independently associated with adverse prognosis (Table 4).

## 4. Discussion

Arterial stenosis or occlusion can lead to hemodynamic changes in brain tissue [23]. As CBF perfusion is affected by various factors like vascular stenosis degree, CC, and cerebrovascular reserve capacity, patients with intracranial artery stenosis or occlusion still have reduced cerebral blood perfusion and vascular reserve even without obvious neurological defects [24]. MCA supplies blood to the whole dorsolateral prefrontal cortex and deep white matter of the cerebral hemisphere, which is widely distributed and easily affected. So MCA stenosis or occlusion in different sites and severity can cause ischemia and hypoxia changes of varying degrees in different parts of the brain tissue, resulting in different clinical manifestations and affecting patients' outcomes [25]. Therefore, early evaluation and intervention can reduce morbidity and improve survival.

3D-ASL is an in vivo perfusion imaging technique using inversion pulses to label water molecules in arterial blood, which can obtain a quantifiable and visualized whole brain perfusion map [26]. According to the recommendation of authorities, this method has superior performance in signal-to-noise ratio, magnetization transfer effect control, and reducing the negative impact of tissue susceptibility which means it is reliable for CBF measurement [18, 27]. CP is a well-recognized and reliable indicator of tissue metabolism and function, as it reflects the blood flow delivered to the unit tissue per unit time [28], while PLD is a very important parameter of 3D-ASL imaging technology, which is defined as the time of signal acquisition from the beginning of labeling of intravascular water molecules to the completion of exchange in the brain [29]. Choi et al. [30] showed that multiple PLDs can evaluate the blood flow reserve capacity after MCAO. Short PLD mainly reflects the behavior characteristics of cerebral vascular perfusion, which indicates the length and thickness of vascular path in the blood supply area. Long PLD, on the other hand, can reflect the final perfusion result, which better shows the blood flow reserve and CC ability. Hence, the selection of PLD is very critical. Maclntosh et al. [31] recommended PLD = 1525 ms when selecting and setting PLD for healthy people. However, for patients with ischemic cerebrovascular disease, the PLD should be slightly extended to 2.0 s because of their poor intracranial vascular condition. And for some patients whose intracranial vascular stenosis degree ≥ 70% have been confirmed by other tests, it makes sense to use a larger PLD time. Therefore, two PLDs (1525 ms and 2525 ms) were used in this study. The results showed that there was a significant difference in CBF of the affected brain between PLD 1525 ms and 2525 ms groups (*P* < 0.05), and the CP volume of the affected cerebral hemisphere in both groups was significantly lower compared with the mirror side (*P* < 0.05). The results are consistent with those of Lin et al. [32], suggesting decreased vascular reactivity. Bokkers et al. [33] also used ASL technique to evaluate CP in patients with internal carotid artery stenosis and found significantly lower CP volume and hemodynamic indexes of the diseased cerebral hemisphere in comparison with the healthy side. In addition, compared with the mirror side, the CBF of the affected side showed hypoperfusion in 43 cases (71.7%), normal perfusion in 9 cases (15.0%), and hyperperfusion in 8 cases (13.3%). CBF in normal and hyperperfused patients decreased when PLD was 1525 ms, but it was returned to normal or increased when PLD was 2525 ms, indicating well compensated CC and good prognosis, which was consistent with Akiyama's research results [34]. Liu et al. [35] found that cerebral tissue perfusion supplied by different vessels on the affected side changes with the aggravation of internal carotid artery stenosis, which mainly depends on the compensatory level of CC blood perfusion.

Then, with the mRS as the outcome indicator, patients were assigned to either the poor or the good prognosis group to study the relationship between long-term prognosis and related factors. The results showed that age, NIHSS score, CC, and Hcy were independent risk factors for poor prognosis. Older patients are less active than younger patients, and their family members are less cooperative, so they are more inclined to stay in bed, resulting in increased complications and decreased rehabilitation effect. Moreover, elderly patients have low organ function, nutritional status, and body resistance, which leads to poor long-term prognosis. This result has been demonstrated in several studies [36]. NIHSS reflects the severity of stroke and provides sound predictive information, becoming an indispensable part of many prediction equations [37]. Singer et al. [38] found that the earlier the onset age and the lower the NIHSS score, the better the prognosis of patients. Good CC has also been shown to significantly reduce cerebral infarction volume in patients with symptomatic intracranial atherosclerotic stenosis and lower the risk of cerebral infarction recurrence [39, 40]. Besides, the degree of CC opening before illness can affect the clinical prognosis of patients more than that after illness [41]. Furthermore, our results suggest that the level of Hcy can also affect the prognosis of patients. Hyperhomocysteinemia can damage blood vessels by causing endothelial damage and dysfunction, affecting smooth muscle cell proliferation, enhancing the expression of inflammatory factors, and inducing oxidative stress mechanisms. Previous evidence also demonstrates a close link between high Hcy levels and stroke severity, poor prognosis, and stroke recurrence. However, this study still has some limitations. First, due to this was a single-center study, the sample size was relatively small; meanwhile, only the patients diagnosed by MRA as unilateral and unilateral middle cerebral artery M1 segment occlusion were included; thus, there might be bias in the selection of patients. Second, empirical hand-drawing was used to select ROI, which was susceptible to the influence of subjective factors of operators. Third, ASL technology can only obtain one related parameter which is CBF, and the research variable is relatively single. Therefore, a large sample size, multicenter study is needed to confirm the results.

To sum up, 3D-ASL is a noninvasive perfusion imaging method, which can intuitively reflect the ischemic perfusion level of the cerebral artery after subtotal occlusion and reduce the CBF of the patient's affected side, providing individual quantitative basis for clinical diagnosis and curative effect evaluation. For patients with unilateral MCAO, older age, high NIHSS scores and Hcy levels, and poor CC are not conducive to prognosis.

## Figures and Tables

**Table 1:** Comparison of CBF between the affected side and the contralateral side in patients with unilateral MCAO.

	Postlabeling delay	CBF of the affected side (mL/(100 g·min))	CBF of the mirror side (mL/(100 g·min))	*t*	*P*
Left MCAO (*n* = 27)	1525 ms	22.95 ± 9.69	44.39 ± 10.21	2.7089	<0.0001
	2525 ms	41.96 ± 10.17^∗^	47.46 ± 9.68	2.7021	0.0469

Right MCAO (*n* = 33)	1525 ms	30.17 ± 11.38	48.08 ± 10.08	2.6464	<0.0001
	2525 ms	44.13 ± 10.52^∗^	50.24 ± 9.57	2.4757	0.0163

Unilateral MCAO (*n* = 60)	1525 ms	27.00 ± 11.36	46.50 ± 10.11	9.9325	<0.0001
	2525 ms	43.15 ± 9.78^∗^	49.28 ± 9.87	3.4173	0.0008

Note: ^∗^*P* < 0.05 vs. ipsilateral 1525 ms group; CBF: cerebral blood flow; MCAO: middle cerebral artery occlusion.

**Table 2:** Comparison of perfusion status between the affected side and mirror side of patients with unilateral MCAO.

rCBF	1525 ms	2525 ms	*t*	*P*
<0.9 (*n* = 43)	25.30 ± 4.88	33.96 ± 5.92	7.4018	<0.0001
0.9-1.1 (*n* = 9)	36.62 ± 2.64	43.71 ± 4.21	4.2803	0.0005
>1.1 (*n* = 8)	50.36 ± 2.96	52.13 ± 1.93	1.4168	0.1784

Note: MCAO: middle cerebral artery occlusion; rCBF: relative cerebral blood flow.

**Table 3:** Comparison of baseline data between the two groups.

Factors	Good prognosis group (*n* = 34)	Poor prognosis group (*n* = 26)	*t*/*χ*^2^	*P*
Sex (male/female)	19/15	15/11	0.0197	0.8885
Age (years old)	54.00 ± 6.88	57.69 ± 6.55	2.7479	0.0080
Location of vascular occlusion			0.0247	0.8752
Left middle cerebral artery	15 (44.12)	12 (46.15)		
Right middle cerebral artery	19 (55.88)	14 (53.85)		
Collateral circulation			5.6681	0.0173
Good	21 (61.76)	8 (30.77)		
Poor	13 (38.24)	18 (69.23)		
Smoking history	14 (41.18)	10 (38.46)	0.0453	0.8315
Drinking history	19 (55.88)	16 (61.54)	0.1939	0.6597
History of hypertension	13 (38.24)	11 (42.31)	0.1018	0.7497
History of diabetes	14 (41.18)	9 (34.62)	0.2683	0.6045
History of coronary heart disease	16 (47.06)	12 (46.15)	0.0048	0.9445
NIHSS score (median (lower quartile, upper quartile))	3 (1.11)	8 (4.13)	3.0140	0.0038
Laboratory inspection index				
Total cholesterol (mmol/L)	4.63 ± 1.31	4.54 ± 0.78	0.3104	0.7574
Triglyceride (mmol/L)	2.12 ± 0.94	1.32 ± 0.56	3.8447	0.0003
High-density lipoprotein (mmol/L)	1.09 ± 0.27	1.02 ± 0.20	1.1088	0.2721
Low-density lipoprotein (mmol/L)	2.80 ± 0.59	2.63 ± 0.69	1.0275	0.3084
Platelet count (×109/L)	224.99 ± 30.50	228.68 ± 51.97	0.3442	0.7319
White blood cell count (×109/L)	7.53 ± 1.49	7.37 ± 1.95	0.3605	0.7198
Albumin (g/L)	39.84 ± 5.02	40.35 ± 6.61	0.3399	0.7352
Hemoglobin (g/L)	139.13 ± 14.32	132.51 ± 14.87	1.7453	0.0862
Serum creatinine (mmol/L)	57.75 ± 6.41	58.92 ± 6.85	0.6801	0.4991
Uric acid (*μ*mol/L)	279.18 ± 87.93	251.76 ± 98.48	1.1363	0.2605
Urea (mmol/L)	4.93 ± 1.04	5.22 ± 1.05	1.0659	0.2909
Homocysteine (mmol/L)	17.71 ± 4.06	26.57 ± 9.86	4.7489	<0.0001
Glutamic-pyruvic transaminase	19.81 ± 3.81	20.16 ± 4.77	0.3161	0.7531

**Table 4:** Multivariate Logistic regression analysis of prognosis.

Indicators	*β*	SE	Wald	*P* value	Exp(*B*)	95% CI
Age (continuous variable)	0.197	0.078	6.404	0.011	1.217	1.045-1.418
Collateral circulation (0 = good; 1 = poor)	2.977	1.094	7.401	0.007	19.630	2.299-167.640
NIHSS score (continuous variable)	0.218	0.098	4.942	0.026	1.244	1.026-1.508
Triglyceride (continuous variable)	-1.089	0.629	2.991	0.084	0.337	0.098-1.156
Homocysteine (continuous variable)	0.135	0.059	5.219	0.022	1.144	1.019-1.284
Constant	-15.383	5.498	7.829	0.005	0.000	-

## Data Availability

The labeled dataset used to support the findings of this study are available from the corresponding author upon request.
